# Fachgebietsspezifische Kenntnisse als Voraussetzung für eine effektive Therapie kritisch kranker Patient:innen

**DOI:** 10.1007/s00104-025-02286-z

**Published:** 2025-04-25

**Authors:** Sonja Vonderhagen, Uwe Hamsen, Andreas Markewitz, Ingo Marzi, Gerrit Matthes, Andreas Seekamp, Georg Trummer, Felix Walcher, Christian Waydhas, René Wildenauer, Jens Werner, Wolfgang H. Hartl, Thomas Schmitz-Rixen, Chirurgische Arbeitsgemeinschaft für Intensiv- und Notfallmedizin (CAIN) als Vorsitzende, Deutsche Gesellschaft für Chirurgie (DGCH) als 3. Vizepräsident, Deutsche Gesellschaft für Chirurgie (DGCH) als Generalsekretär

**Affiliations:** 1 Klinik für Unfall‑, Hand- und Wiederherstellungschirurgie, Universitätsklinikum, Universitätsmedizin Essen, Essen, Deutschland; 2 Klinik und Poliklinik für Chirurgie, Berufsgenossenschaftliches Universitätsklinikum Bergmannsheil Bochum, Bochum, Deutschland; 3 Bendorf, Deutschland; 4 Klinik für Unfall‑, Hand- und Wiederherstellungschirurgie, Universitätsklinikum Frankfurt Frankfurt/Main, Frankfurt/Main, Deutschland; 5 Klinik für Unfall- und Wiederherstellungschirurgie, Klinikum Ernst von Bergmann, Potsdam, Deutschland; 6 Klinik für Unfallchirurgie und Orthopädie, Universitätsklinikum Schleswig-Holstein, Campus Kiel, Kiel, Deutschland; 7 Klinik für Herz- und Gefäßchirurgie, Universitäts-Herzzentrum Freiburg-Bad Krozingen und Medizinische Fakultät, Universität Freiburg, Freiburg, Deutschland; 8 Universitätsklinik für Unfallchirurgie, Universitätsmedizin Magdeburg, Magdeburg, Deutschland; 9 Hausarztzentrum Wiesentheid, Wiesentheid, Deutschland; 10 Allgemein-, Viszeral- und Transplantationschirurgie, Klinikum der Universität, Campus Grosshadern, Ludwig-Maximilians-Universität München, Marchioninistr. 15, 81377 München, Deutschland; 11 Klinik für Gefäß- und Endovaskularchirurgie, Goethe-Universität Frankfurt am Main, Frankfurt am Main, Deutschland; 12 Deutsche Gesellschaft für Chirurgie e. V., Langenbeck-Virchow-Haus, Luisenstr. 58/59, 10117 Berlin, Deutschland

**Keywords:** Intensivmedizin, Weiterbildung, Zusatzweiterbildung, Fachgebiet, Sepsis, Infektion, Inflammation, Chirurgie, Intensive care, Primary specialty training, Supra-specialty training, Medical specialty, Sepsis, Infection, Inflammation, Surgery

## Abstract

In Deutschland ist seit dem letzten Deutschen Ärztetag im Mai 2024 eine Diskussion über die Verkürzung der Facharztweiterbildung und eine Verlagerung von Inhalten einer Zusatzweiterbildung in die bisherige Facharztweiterbildung entstanden. Dies betrifft auch die Intensivmedizin, mit der Perspektive, einen Facharzt für fachgebietsspezifische Intensivmedizin (z. B. Facharzt für chirurgische Intensivmedizin) zu schaffen. Die damit einhergehende Reduktion allgemeiner, fachgebietsspezifischer Inhalte halten wir aus mehreren Gründen für nicht sachgerecht: Die Kenntnis der fachgebietsspezifischen Auslösefaktoren („Foci“) einer kritischen Erkrankung (Organdysfunktion) sowie die Kenntnis der jeweiligen, auslösefaktorenspezifischen Symptomatik, Diagnostik und Abläufe zur Einleitung einer kausalen Therapie sind entscheidend für die Prognose. Neueste Erkenntnisse weisen darauf hin, dass bei septischen Foci eine Zeitspanne zwischen Diagnosestellung und Fokustherapie von ca. 6 h nicht überschritten werden sollte, um eine Verschlechterung der Prognose zu vermeiden. Um die Zeit zwischen Symptombeginn und effektiver Therapie der Auslösefaktoren nicht zu lang werden zu lassen, ist eine vertiefte fachspezifische Kompetenz im gesamten Prozess erforderlich. Diese Kompetenz ist unabhängig von der intensivmedizinischen Qualifikation und kann nur im Rahmen einer ausreichenden, fachgebietsspezifischen Weiterbildung (mit darauffolgender, intensivmedizinischer Zusatzweiterbildung) erworben werden. Fachgebietsspezifische Kenntnisse sind Voraussetzung für eine effektive Therapie kritisch kranker Patient*innen. Die Beibehaltung der bisherigen fachgebietsspezifischen Weiterbildung und der damit verbundene Erwerb spezifischer Kenntnisse im jeweiligen Fachgebiet ermöglichen es ferner, Fachärzt:innen im Klinikbetrieb breiter einsetzen zu können sowie diagnostische und therapeutische Ressourcen schonender zu verwenden. Die intensivmedizinische Zusatzweiterbildung sollte nicht zulasten fachgebietsspezifischer Inhalte gehen und muss in der nächsten Änderung der Weiterbildungsordnung weiter von allen Facharztqualifikationen des Gebietes Chirurgie heraus erreichbar bleiben. Aufgrund des unumgänglichen Umfangs kann die intensivmedizinische Zusatzweiterbildung selbst nur hauptberuflich und die gesamte Arbeitszeit einnehmend erfolgen.

## Abstract (en)

Since the last meeting of the German Medical Association in May 2024, there has been a discussion in Germany about the shortening of primary specialty training and a transfer of the contents of additional supra-specialty training to the existing primary specialty training. This also affects intensive care medicine, with the prospect of creating a subspecialty for subspecialties in intensive care medicine (e.g., a specialty in surgical intensive care medicine). We consider the associated reduction of general specialty-specific contents to be inappropriate for several reasons. Knowledge of the specialty-specific trigger factors (foci) of a critical illness (organ dysfunction) as well as knowledge of the respective trigger factor-specific symptoms, diagnostics and pathways for initiating a causal treatment, are decisive for the prognosis. Recent evidence suggests that in the case of septic foci a time span between making the diagnosis and treatment of the focus should not exceed ca. 6h in order to avoid a worsening of the prognosis. To ensure that the time between symptom onset and effective treatment of the causal factors is not too long, an in-depth expertise in the primary specialty is required throughout the process. This expertise is independent of training in intensive care medicine and can only be acquired through adequate training in the specialty, followed by additional training in intensive care medicine. Expertise in the primary specialty is a prerequisite for the effective treatment of critically ill patients. Maintaining the training specific to the primary specialty and the associated acquisition of specific knowledge in the respective specialty also enables a wider deployment of specialists in clinical practice and a more economical use of diagnostic and therapeutic resources. The additional training in intensive care medicine (supraspecialty) should not be at the expense of content specific to the primary specialty and must remain accessible to all surgical specialties in the field of surgery in the next revision of the training regulations. Due to the unavoidable extent, the additional training in intensive care medicine can itself only be provided on a full-time basis.

## Hintergrund

In Europa existieren derzeit drei verschiedene Zugangsmöglichkeiten zur intensivmedizinischen Weiterbildung:

- der exklusive Facharzt für Intensivmedizin (Spanien, Schweiz, Vereinigtes Königreich),
- der fachgebietsspezifische Facharzt mit intensivmedizinischer Zusatzweiterbildung (z. B. der Facharzt für Chirurgie mit intensivmedizinischer Zusatzweiterbildung) sowie – teilweise nebeneinander –
- der Facharzt für fachgebietsspezifische Intensivmedizin (z. B. der Facharzt für internistische Intensivmedizin, teilweise jedoch exklusiv für Anästhesisten mit Intensivmedizin; Tab. 1 [1]).

In den USA besteht nur ein Zugang zur Intensivmedizin über den fachgebietsspezifischen Facharzt mit anschließender intensivmedizinischer Zusatzweiterbildung („fellowship specialty training and board certification in critical care medicine“ [2]).

**Tab. 1:** Zugangsmöglichkeiten zur intensivmedizinischen Weiterbildung in Europa. (Nach [1])

Weiterbildungsmöglichkeiten		Länder
Zugang zur intensivmedizinischen Weiterbildung	Facharzt für Intensivmedizin	Spanien^a^
		Schweiz
		Vereinigtes Königreich^b^
	Supraspezialität	Fachgebietsspezifischer Facharzt mit Zusatzweiterbildung: 16 von 33 Ländern
	Subspezialität	Nur im Fach Anästhesie: 13 von 33 Ländern (Facharzt für anästhesiologische Intensivmedizin)
		Verschiedene Fachdisziplinen: 5 von 33 Ländern (Facharzt für fachgebietsspezifische Intensivmedizin)
Weiterbildungsprogramm	Dauer	Supraspezialität: 24 Monate (14 von 33 Ländern)
		Subspezialität: 6–24 Monate (während der anästhesiologischen Facharztausbildung, Spannweite: 36–72 Monate)
		Facharzt für Intensivmedizin: 32–72 Monate

Die vorgelegten Daten beziehen sich auf 31 Länder in Europa und auf Daten aus der Türkei und Israel: Österreich, Belgien, Bulgarien, Kroatien, Zypern, Tschechische Republik, Dänemark, Estland, Finnland, Frankreich, Georgien, Deutschland, Griechenland, Ungarn, Irland, Island, Italien, Lettland, Litauen, Malta, Niederlande, Norwegen, Polen, Portugal, Slowakei, Slowenien, Spanien, Schweden, Schweiz und das Vereinigte Königreich

^a^Facharzt für anästhesiologische Intensivmedizin möglich

^b^Facharzt für Intensivmedizin zusätzlich zum Facharzt für Anästhesie, Notfallmedizin, Innere Medizin und Chirurgie möglich

In Europa wurde die Intensivmedizin im Rahmen der europäischen Richtlinie zur Anerkennung von Berufsqualifikationen bisher nicht als primäres Fachgebiet anerkannt. Eine akute und erhöhte Nachfrage nach Intensivmedizin aufgrund der COVID-19-Pandemie nahm jedoch die Europäische Gesellschaft für Intensivmedizin (European Society of Intensive Care Medicine [ESICM], die allerdings nur einen kleinen Teil aller Intensivmediziner in ganz Europa vertritt) zum Anlass, 2021 eine Initiative zu Reformierung der Weiterbildung zu starten. Ziel war (und ist) es, die Weiterbildung zu verkürzen und die Freizügigkeit für Intensivmediziner in Europa zu fördern. Vorgeschlagen wurde die Anerkennung der Intensivmedizin in Europa als eigenständiges medizinisches Fachgebiet gemäß Anhang V der Europäischen Richtlinie über die Anerkennung von Berufsqualifikationen (Richtlinie 2005/36/EG des Europäischen Parlaments und des Rates [3]).

In den deutschen Bundesländern steht seit der letzten Änderung der Musterweiterbildungsordnung (gültig seit 2018) für alle Kernfächer wie Neurologie, Chirurgie, Innere Medizin, Pädiatrie, Neurochirurgie und Anästhesie ausschließlich eine intensivmedizinische Zusatzweiterbildung offen (fachgebietsspezifischer Facharzt mit intensivmedizinischer Zusatzweiterbildung [4, 5]).

Aktuell ist jedoch auch in Deutschland eine Diskussion um die Änderung der Facharztweiterbildung und deren Bezug zur intensivmedizinischen Zusatzweiterbildung entstanden – ähnlich zu dem von der ESICM angestoßenen Prozess. Auf dem Deutschen Ärztetag 2024 erteilten die Delegierten einen Auftrag an die Ständige Konferenz (StäKo) „Ärztliche Weiterbildung“ der Bundesärztekammer, die (Muster‑)Weiterbildungsordnung (MWBO) zu entschlacken und für die Gebiete, Facharztweiterbildungen und Schwerpunkte die Weiterbildungszeiten zu prüfen und ggf. zu reduzieren. Ferner ist angedacht, die Zusatzweiterbildungen von ihrer Ausgestaltung her präziser zu charakterisieren [5]:

- Stufe C1: Zusatzweiterbildung hauptberuflich und die gesamte Arbeitszeit einehmend,
- Stufe C2: berufsbegleitenden Zusatzweiterbildung ohne eine vorgegebene Weiterbildungszeit und ohne den Aspekt der „Hauptberuflichkeit“,
- Stufe C3: Zusatzweiterbildung durch Kurse nach Weiterbildungsordnung mit Lernerfolgskontrolle, aber ohne Weiterbildungszeiten, und ohne Weiterbildung bei einem oder einer Weiterbildungsbefugten.

Aktuelle Überlegungen der StäKo gehen inzwischen tatsächlich dahin, generell die Weiterbildungszeiten zu verringern (auf ein Maximum von fünf Jahren für den Fachgebiets-spezifischen Facharzt (z. B. Facharzt für Viszeralchirurgie) [5, 6]). Im Zusammenhang mit diesen Verkürzungen wird auch eine Umwandlung des intensivmedizinisch weitergebildeten fachgebietsspezifischen Facharztes (z. B. Facharzt für Chirurgie mit intensivmedizinischer Zusatzweiterbildung) in einen Facharzt für fachgebietsspezifische Intensivmedizin (z. B. Facharzt für chirurgische Intensivmedizin) diskutiert (mit verkürzter Weiterbildungszeit durch Reduktion der nichtintensivmedizinischen Inhalte in der alten fachgebietsspezifischen Facharztweiterbildung und ggf. mit Abstufung der Zusatzweiterbildung von Kategorie C1 zu C2). Schon auf dem Deutschen Ärztetag 2025 könnten dann entsprechende „Entschlackungsmaßnahmen“ beschlossen werden. Die Fachgesellschaften sind zurzeit aufgefordert, hierzu Konzepte zu entwickeln und der StäKo zu empfehlen.

Im Folgenden soll dargestellt werden, warum die unter dem Dach der Deutschen Gesellschaft für Chirurgie (DGCH) versammelten, operativ tätigen Fachgebiete eine solche Umwandlung mit Reduktion fachgebietsspezifischer Inhalte nicht befürworten, sondern für eine Beibehaltung der aktuellen Weiterbildung plädieren (fachgebietsspezifischer Facharzt mit hauptberuflicher intensivmedizinischer Zusatzweiterbildung): Die Begründung dafür liegt einerseits im unumgänglichen Umfang der intensivmedizinischen Zusatzweiterbildung und andererseits in der Komplexität kritisch kranker Patient:innen und den sich daraus ergebenden Konsequenzen für die Behandlung fachgebietsspezifischer Erkrankungen, die wiederum Ursache der (oft lebensbedrohlichen) sekundären Organdysfunktion sind. In der Folge wird für diese Behandlung gezeigt werden, dass Diagnose und Therapie fachgebietsspezifischer Erkrankungen (und die damit assoziierten Zeitfenster) die prognostisch entscheidenden Determinanten darstellen.

### Kernaussage 1.

Die intensivmedizinische Weiterbildung ist integraler Bestandteil der chirurgischen Tätigkeiten, und eine frühe Einbindung und intensivmedizinische Erfahrung der Weiterbildungsärzte muss gewährleistet bleiben. Die Vorverlagerung der intensivmedizinischen Zusatzweiterbildung in den (ggf. zusätzlich reduzierten) Zeitabschnitt der Facharztweiterbildung würde zulasten fachgebietsspezifischer Weiterbildungsinhalte gehen.

## Die Komplexität der kritischen Krankheit

**Abb. 1:**
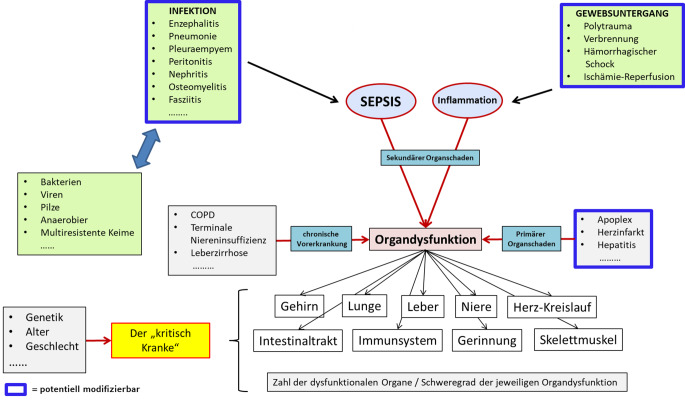
Die Dimensionen der kritischen Krankheit

Der/die als Intensivpatient:in bezeichnete Patient:in ist eine Chimäre, und die zugehörige „kritische Krankheit“ stellt eine extrem heterogene Mischung verschiedenster fachgebietsspezifischer Charakteristika dar bezogen auf Grunderkrankung, Komorbidität und Organdysfunktion (inklusive der diese auslösenden Mechanismen; Abb. 1). Konzeptionell kann sich eine Intensivpflichtigkeit ergeben aus (a) hohem Pflegebedarf, (b) hohem Überwachungsbedarf und (c) hohem Bedarf an organfunktionsunterstützender – und/oder ersetzender Therapie. Im Folgenden soll speziell letzterer Fall betrachtet werden.

**Abb. 2:**
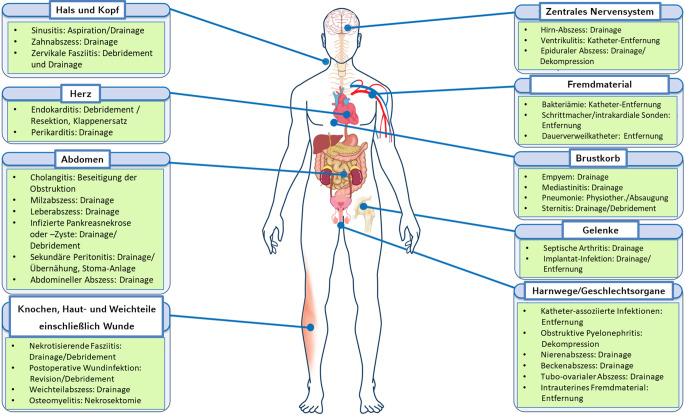
Lokalisation entzündlicher Foci als Auslöser einer Organdysfunktion und deren kausale, fachgebietsspezifische Therapiemöglichkeiten. (Nach [34])

Eine intensivpflichtige Organdysfunktion ist entweder Folge einer primären Organerkrankung (wie Herzinfarkt, Hepatitis, Apoplex), oder die Dysfunktion entwickelt sich sekundär über exazerbierende immunologische Reaktionen im Rahmen von Infektion (Sepsis) bzw. Inflammation. Infektionen und nichtinfektiöse Inflammationen können verschiedenste Ursachen haben und praktisch alle Regionen/Kompartimente des Körpers betreffen (Abb. 2; Tab. 2). Ebenfalls zahlreich ist die Qualität der Mikroorganismen, die Infektionen auslösen können. In Abhängigkeit vom betroffenen Organ ist die sekundäre Organdysfunktion dabei durch eine spezifische Funktionsstörung gekennzeichnet, die unterschiedlichste Schweregrade annehmen kann und im Überlebensfall u. U. mit charakteristischen Langzeitschäden verbunden ist (Tab. 3 [7, 8]).

**Tab. 2:** Nichtentzündliche Foci als Auslöser einer Organdysfunktion und deren kausale Therapiemöglichkeiten

Nichtentzündliche Foci	Kausale Therapie
Trauma (Frakturen/Weichteilschaden/Kompartmentsyndrom)	Reposition/Stabilisierung/Dekompression
Verbrennung	Débridement/Nekrosektomie
Gesamtkörperischämie/Reperfusion (hämorrhagischer/kardiogener Schock)	Aggressive Blutstillung/Transfusion/Reanimation/Volumenmanagement
Regionale Ischämie/Reperfusion	Revaskularisierung

**Tab. 3:** Inflammatorisch induzierte Organdysfunktion und zugehörige Pathomechanismen, symptomatische Therapien sowie Langzeitfolgen

Organsystem	Art der Dysfunktion	Dominierender Pathomechanismus	Grundzüge der symptomatischen Therapie	Mögliche Langzeitfolgen
ZNS	Septische/inflammatorische Enzephalopathie	Intrazerebrale Zytokinexposition	Sedativa/Neuroleptika	Kognitive Defizite
Peripheres Nervensystem	Axonaler Schaden	Mikrozirkulationsschaden^a^ im Epineurium	Physiotherapie	„Critical illness polyneuropathy“
Lunge	ARDS	Pulmonal-arterieller Mikrozirkulationsschaden^a^	(nicht-)invasive mechanische Beatmung, Lagerungstherapie, VV-ECMO	Lungenfibrose
Leber	Cholangitis	Mikrozirkulationsschaden^a^ an den Gallengängen	–	Sekundär sklerosierende Cholangitis
Niere	Akutes Nierenversagen	Mikrozirkulationsschaden^a^ im Tubulussystem	Mechanische Nierenersatztherapie	Chronische Niereninsuffizienz
Herz/Kreislauf	Distributiver Schock, Kardiomyopathie	Mikrozirkulationsschaden^a^ im Subkutan‑/Fettgewebe, kardiale Zytokinexposition	Katecholamine/intravenöse Flüssigkeitszufuhr/Steroide, mechanische Herz-Kreislauf-Unterstützung (z. B. IABP, VA-ECMO)	Chronische Hypotonie
Intestinaltrakt	Paralyse/Distension/Translokation	Intestinaler Mikrozirkulationsschaden^a^	Properistaltika/Dekontamination/Resektion/Stoma	–
Immunsystem	Hyperinflammation (unspezifisches Immunsystem), sekundäre Immunsuppression (spezifisches Immunsystem)	Inflammatorische Mediatoren aus Granulozyten/Makrophagen	Beseitigung des primären Auslösemechanismus	Chronische Immunschwäche
Gerinnung	Disseminierte intravaskuläre Koagulation/Mikrothrombosierung	Inflammatorische Mediatoren aus Granulozyten/Makrophagen	Niedermolekulares Heparin/Thrombinhemmung/Gerinnungsfaktorengabe	–
Muskuloskelettales System	Muskeleiweißkatabolie	Muskuläre Zytokin‑/Kortisolexposition, Immobilisation	Medizinische Ernährungstherapie, Physiotherapie	„Critical illness myopathy“, „ICU-acquired weakness“

*ECMO* extrakorporale Membranoygenierung, *VV* venovenös, *VA* venoarteriell, *IABP* intraaortale Ballonpumpe, *ICU* Intensive Care Unit

^a^Der Mikrozirkulationsschaden umfasst auf der arteriellen Seite ein kapilläres Leck mit Verlängerung der interstitiellen O_2_-Diffusionsstrecke und auf der venösen Seite eine Mikrothrombosierung mit kapillärer Perfusionseinschränkung

**Abb. 3:**
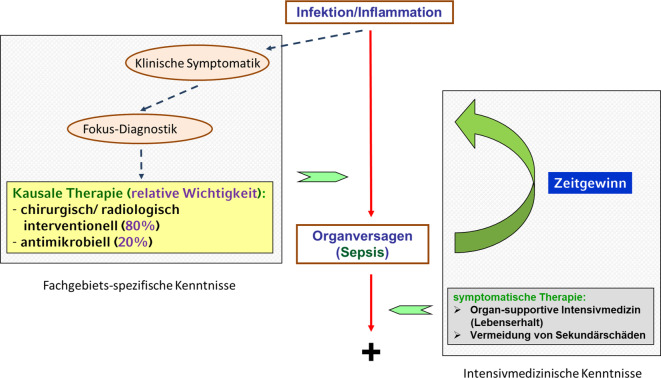
Therapeutische Komponenten bei der Therapie von Infektion und Inflammation

Die Behandlung der Organdysfunktion beinhaltet zwei Komponenten, einmal die fachgebietsspezifische kausale Therapie und dann die supportive, nicht notwendigerweise fachgebietsspezifische symptomatische Intensivtherapie (Abb. 3). Diese beiden Komponenten sind additiv (und nicht etwa komplementär), besitzen jedoch grundsätzlich unterschiedlichen Charakter. Während erstere ihr primäres Ziel in der Heilung bzw. Ausschaltung des ursächlichen Fokus sieht, steht bei letzterer die Sicherung des Überlebens und die Vermeidung sekundärer Folgeschäden im Mittelpunkt. Zuletzt wurden 2022 die Inhalte der intensivmedizinischen Zusatzweiterbildung von der Bundesärztekammer definiert [9], diese Inhalte sollen hier nicht weiter diskutiert werden. Im Folgenden soll näher auf die Bedeutung des fachgebietsspezifischen Wissens eingegangen werden. Die fachgebietsspezifische Genese der Organdysfunktion bedingt, dass eine effiziente kausale Therapie der Auslösefaktoren („Foci“, Abb. 2; Tab. 2) ein vertieftes, fachgebietsspezifisches Wissen über eben diese voraussetzt.

### Kernaussage 2.

Die Behandlung der intensivpflichtigen Organdysfunktion beinhaltet zwei Komponenten, einmal die fachgebietsspezifische kausale Therapie und dann die supportive, nicht notwendigerweise fachgebietsspezifische symptomatische Intensivtherapie. Diese beiden Komponenten sind additiv (und nicht etwa komplementär), besitzen jedoch grundsätzlich unterschiedlichen Charakter.

## Der fachgebietsspezifische Krankheitsfokus und seine Bedeutung für die Intensivtherapie

Die Zahl der fachgebietsspezifische Foci, die eine intensivpflichtige Organdysfunktion auslösen können, ist groß (Abb. 2), woraus eine enorme Heterogenität alleine aus Sicht der anatomischen Lokalisation resultiert. Diese fachgebietsspezifische Fokusheterogenität hat es – in Verbindung mit der ausgeprägten Komplexität der Organdysfunktion – bis zum heutigen Tag außerordentlich erschwert, allgemein gültige Empfehlungen zur Diagnostik, Therapie und zum allgemeinen Management kritisch kranker Patient*innen auf der Basis hochwertiger wissenschaftlicher Studien (mit klaren, therapeutisch relevanten Ergebnissen) zu formulieren. Exemplarisch lässt sich z. B. für infektiöse Krankheitsbilder feststellen, dass in der Deutschen Sepsisleitlinie von 2018 für 109 Themenkomplexe bei 67,0 % der Fragen oder Problemen entweder keine Empfehlung abgegeben werden konnte oder die Empfehlungen nur auf Expertenkonsens bzw. niedrigem bzw. sehr niedrigem Evidenzgrad beruhten. Nur 6,4 % der Empfehlungen hatten einen hohen Evidenzgrad als Grundlage; [10]. Bei den (aktuelleren) Leitlinien der Surviving Sepsis Campaign (2021) zum Management von Sepsis und septischem Schock finden sich noch ungünstigere Prozentsätze (76,3 %, respektive 3,2 % [11]). Durchweg negative Ergebnisse erbrachten bisher alle Studien zur gezielten adjuvanten medikamentösen Therapie des ARDS und der Sepsis [12, 13]. Selbst scheinbar einfache Sachverhalte wie die Diagnose einer Sepsis/eines septischen Schocks sind dadurch kompliziert, dass bis heute kein praktikables Screeninginstrument zu Verfügung steht, das höchsten Ansprüchen an Sensitivität und Spezifität genügen würde (einschließlich des sog. qSOFA-Scores [11]).

Derzeit gibt es somit extrem wenig gesichertes therapeutisches Wissen auf der Basis hochwertiger klinischer Studien, und ein Großteil der Diagnostik und Therapie fachgebietsspezifischer Infektion und nichtinfektiöser Inflammation hängt weiterhin zum größten Anteil von der individuellen Kompetenz und Erfahrung des behandelnden Arztes im jeweiligen Fachgebiet ab.

## Die relative Wichtigkeit therapeutischer Maßnahmen bei der Versorgung kritisch kranker Patient:innen

Im Folgenden soll nur die sekundäre Organdysfunktion betrachtet werden. Hier besteht sehr häufig eine naturinhärente Chance auf Restitutio ad Integrum, wenn die Ursache eliminiert wurde. Im Mittelpunkt der therapeutischen Bemühungen steht somit nicht nur, das Auftreten einer neuen Organdysfunktion bzw. die Verschlechterung einer bereits bestehenden Organdysfunktion zu verhindern, sondern dem Organismus auch die Chance auf eine spontane Erholung zu geben. Um diese Chance wahrnehmen zu können, muss als Grundvoraussetzung eine kausale, fachgebietsspezifische Therapie erfolgen, die begleitet wird von einer supportiven Therapie und Kompensation (potenziell) lebensbedrohlicher Organdysfunktionen.

Die Möglichkeiten zur ursächlichen anatomisch bzw. pathophysiologisch begründeten Therapie hängen hochkomplex von den Charakteristika des auslösenden Fokus ab und sind fachspezifisch (Abb. 2; Tab. 2); ähnlich komplex sind die Verfahren zur organsupportiven symptomatischen Therapie, die organspezifisch mechanische oder medikamentöse Komponenten beinhaltet (Tab. 3). Eine effiziente kausale Therapie setzt ferner ein sicheres Erkennen der spezifischen Symptomatik und eine zuverlässige Diagnose des ursächlichen Fokus (infekt- oder nichtinfektinduzierte Inflammation) voraus (Abb. 3).

Die Wichtigkeit der therapeutischen Maßnahmen zueinander ergibt sich aus der Hierarchie prognoserelevanter Variablen (z. B. im Hinblick auf das Überleben). Prognostisch relevant sind zahlreiche Entitäten, die entweder nicht modifizierbar (z. B. Alter, Geschlecht, Komorbidität) oder modifizierbar sind. Modifizierbare Variablen beinhalten – neben der Therapie der primären Organdysfunktion (z. B. Myokardinfarkt) – die Therapie der Auslöser einer sekundären Organdysfunktion (Abb. 2; Tab. 2) bzw. die Vemeidung von Nebenwirkungen einer inadäquaten organsupportiven Therapie (z. B. Beatmung mit hohen Tidalvolumina).

Erst seit kurzem ist es möglich, prognostisch relevante Variablen von ihrer Wichtigkeit her einzuordnen. Untersucht wurde diese Fragestellung im Rahmen prospektiver Beobachtungsstudien mit niedrigem statistischen Bias am Symptom der nosokomialen oder ambulant erworbenen Infektion, Sepsis oder Bakteriämie (Tab. 4). Unter Federführung der ESICM wurde die bisher größte multinationale Studie speziell zur abdominellen Sepsis (AbSeS, Abdominal Sepsis Study: Epidemiology of Etiology and Outcome) durchgeführt. Zentrales Ergebnis war, dass bei abdominellem Fokus eine innerhalb von 7 Tagen nach Diagnosestellung nicht erfolgreiche anatomische Fokuskontrolle die 28-Tages-Sterblichkeit etwa um das 4‑ bis 5‑Fache signifikant erhöhte und dass diese Ineffizienz die bei weitem stärkste prädiktive Variable war [14–16]. Eine erfolgreiche Fokuskontrolle wiederum war der Faktor, der die Prognose am stärksten verbesserte [17, 18].

**Tab. 4:** Beobachtungsstudien mit niedrigem statistischen Bias, die die Effizienz der Fokuskontrolle auf die Sterblichkeit von Patient:innen mit Infektion, Sepsis oder Bakteriämie untersuchten

Erstautor	Charakteristika	Effizienz der Fokuskontrolle^a^	Assoziation mit 28-Tages-Sterblichkeit
Arvaniti K [14]	Kritisch Kranke mit ambulant erworbener oder nosokomialer abdomineller Infektion, Alter ≥ 40 Jahre (*n* = 2337)	Innerhalb von 7 Tagen *nicht *erfolgreich	aOR 5,20
			95 %-CI 4,14–6,54
De Pascale G [15]	Kritisch Kranke mit nosokomialer abdomineller Infektion (*n* = 1077)	Innerhalb von 7 Tagen *nicht* erfolgreich	aOR 5,70
			95 %-CI 3,99–8,18
Blot S [16]	Kritisch Kranke mit ambulant erworbener oder nosokomialer abdomineller Infektion (*n* = 2850)	Innerhalb von 7 Tagen *nicht* erfolgreich	aOR 4,85
			95 %-CI 3,79–6,22
Rüddel H [17]	Kritisch Kranke mit ambulant erworbener oder nosokomialer Sepsis und mit Indikation zur chirurgischen Fokuskontrolle (*n* = 1563)	Erfolgreich	aOR 0,12
			95 %-CI 0,08–0,16
Tabah A [18]	Kritisch Kranke mit nosokomialer Bakteriämie und mit Indikation zur Fokustherapie (*n* = 2600)	Erfolgreich	aOR 0,63
			95 %-CI 0,52–0,76

*aOR* adjustiertes Odds Ratio, *CI* „confidence interval“

^a^Nach Diagnosestellung

Somit ist die fachgebietsspezifische Diagnostik und Therapie des entzündlichen Fokus, der die sekundäre Organdysfunktion auslöst, die prognostisch entscheidende Determinante. Auch wenn keine Studien zu nichtentzündlichen Foci vorliegen, so muss auf der Basis der starken konzeptionellen Ähnlichkeiten davon ausgegangen werden, dass auch bei diesen Foci eine vergleichbare Wichtigkeit der fokusspezifischen anatomischen Therapien besteht (Tab. 2).

### Kernaussage 3.

Die fachgebietsspezifische Diagnostik und Therapie des die sekundäre Organdysfunktion auslösenden Fokus ist die prognostisch entscheidende Determinante.

## Die Bedeutung der Zeitachse bei der Fokustherapie

In konservativen Fachgebieten (Neurologie, Innere Medizin) ist die große Bedeutung der Zeitachse zur Therapie einer primären Organdysfunktion seit langem bekannt. Für z. B. Apoplex oder Myokardinfarkt wurden in den letzten Jahren klar umrissene Zeitfenster festgelegt, deren Einhaltung Voraussetzung für eine effektive Therapie ist [19, 20].

Bei sekundärer Organdysfunktion als Folge von hoch akuten inflammatorischen Foci (z. B. Ischämie-Reperfusion) ist aus pathogenetischer Sicht die Bedeutung einer maximal schnellen Symptomerkennung, Diagnostik und Therapie ebenfalls offensichtlich und benötigt keiner zusätzlichen wissenschaftlichen Begründung. Auch bei weniger akuten inflammatorischen (z. B. ausgeprägtes Weichteiltrauma) oder entzündlichen (z. B. Peritonitis) Krankheitsbildern sollte allein aus pathophysiologischen Überlegungen die schnellstmögliche Elimination des ursächlichen Fokus prognostisch relevant sein. Die Berücksichtigung der Zeitachse ist somit zentraler Bestandteil der kausalen fachgebietsspezifischen Therapie und wird z. B. im Zusammenhang mit entzündlichen Krankheitsbildern auch in allen Leitlinien gefordert [10, 11]. Für diese Krankheitsbilder war es bisher jedoch nicht möglich gewesen, den entscheidenden, optimalen Zeitrahmen speziell zwischen Diagnose und Therapie zuverlässig zu definieren.

Mehrere große prospektive Beobachtungsstudien (einschließlich der AbSeS Studie) an Patient:innen mit nosokomialer oder ambulant erworbener Sepsis/abdomineller Infektion/perforiertem Ulkusleiden und mit Indikation zur Fokuskontrolle erlauben inzwischen zumindest für entzündliche Krankheitsbilder die Bestimmung eines präziseren Zeitraums (Tab. 5). Da in diesen Studien aus ethischen Gründen eine Randomisierung nicht möglich war, kann zwar nur von Assoziationen gesprochen werden. Falls jedoch die analytische Strategie einer beobachtenden Studie der einer randomisierten Studie ähnelt, kann mit hoher Wahrscheinlichkeit eine Kausalität angenommen werden [21].

**Tab. 5:** Beobachtungsstudien mit niedrigem statistischen Bias, die den Einfluss der Zeitdauer zwischen Diagnosestellung und Kontrolle der Infektionsquelle auf die Sterblichkeit von Patient:innen mit Sepsis und septischem Schock untersuchten

Erstautor	Charakteristika	Definition der frühen Fokuskontrolle^a^	Assoziation mit Sterblichkeit
De Pascale G [15]	Kritisch Kranke mit nosokomialer abdomineller Infektion (*n* = 1077)	2–6 h	Frühzeitige vs. verzögerte Fokuskontrolle: aOR 0,50, 95 %-CI: 0,34–0,73
Reitz KM [22]	Ambulant erworbene Sepsis (Sepsis-3) mit Indikation zur Fokuskontrolle (*n* = 4962)	< 6 h	Frühzeitige vs. verzögerte Fokuskontrolle: aOR 0,71, 95 %-, CI: 0,63–0,80
Rüddel H [17]	Kritisch Kranke mit ambulant erworbener oder nosokomialer Sepsis und mit Indikation zur chirurgischen Fokuskontrolle (*n* = 1563)	< 6 h	Verzögerte vs. frühzeitige Fokuskontrolle: aOR 1,64, 95 %-CI: 1,01–2,67
Boyd-Carson H [23]	Kranke mit Indikation zur Notfalllaparotomie bei perforiertem Ulkusleiden (*n* = 3809)	Schnellstmöglich	Pro h Verzögerung: aOR 1,04, 95 %-CI: 1,02–1,07
Martínez ML [24]	Kritisch Kranke mit Sepsis ± septischem Schock (*n* = 1090)	< 12 h	Verzögerte vs. frühzeitige Fokuskontrolle: aOR 1,08, 95 %-CI: 0,76–1,59

*aOR* adjustiertes Odds Ratio, *CI* „confidence interval“, *RR* relatives Risiko

^a^Nach Diagnosestellung

In Gesamtschau aller Ergebnisse scheint das kritische Zeitfenster für eine effektive Sanierung entzündlicher Foci bei etwa 6 h nach Diagnosestellung zu liegen. Die Elimination des Fokus nach dieser Zeitspanne war mit einer signifikanten Prognoseverschlechterung verbunden ([15, 17, 22–24]; Tab. 5).

Neben der ursachenspezifischen (anatomischen) Therapie entzündlicher Foci (Abb. 3) ist die adäquate Antibiose die zweite Säule der kausalen Infekttherapie. Obwohl schon seit längerem ein Zusammenhang zwischen dem Zeitpunkt des Beginns der Antibiose und der Prognose vermutet wurde, war doch die zugrunde liegende Studienqualität ein Kritikpunkt (teilweise hoher statistischer Bias der Beobachtungsstudien). Inzwischen liegen Studien mit niedrigem Bias und sehr hoher Fallzahl (5- bis 6‑stellig) vor, die einen eindeutigen Zusammenhang dokumentieren (Tab. 6). Gerade bei Hochrisikokonstellationen (septischer Schock) scheint es prognoserelevant zu sein, eine mutmaßlich resistenz-/pathogengerechte Antibiose so schnell wie möglich nach Diagnosestellung zu applizieren [25, 26]. Bei geringer ausgeprägter Organdysfunktion scheint ein Zeitfenster von 3–6 h auszureichen [17, 26, 27]. Die aktuellen Studien lassen aber auch erkennen, dass die prognostische Relevanz der adäquaten Antibiotikatherapie um den Faktor 4 bis 5 unter der der anatomischen Therapie liegt [14, 17].

**Tab. 6:** Beobachtungsstudien mit niedrigem statistischen Bias, die den Einfluss der Zeitdauer zwischen Diagnosestellung und dem Beginn einer adäquaten Antibiose auf die Sterblichkeit von Patient:innen mit Sepsis und septischem Schock untersuchten

Erstautor	Charakteristika	Definition des frühen Beginns einer Antibiose^a^	Auswirkung auf Sterblichkeit
Yang A [25]	Kranke mit ambulant erworbener Sepsis (Sepsis-2) ± septischem Schock (*n* = 55.169)	Schnellstmöglich	Pro h Verzögerung: aOR 1,03, 95 %-CI: 1,02–1,04
Hechtman RK [27]	Kranke mit ambulant erworbener Sepsis (> 2 SIRS-Kriterien) (*n* = 273.255)	≤ 3 h	Früherer vs. verzögerter Beginn der Antibiose: RR 0,91, 95 %-CI: 0,89–0,93
Rüddel H [17]	Kritisch Kranke mit ambulant erworbener oder nosokomialer Sepsis, und mit Indikation zur chirurgischen Fokuskontrolle (*n* = 1563)	< 6 h	Verzögerter vs. früherer Beginn der Antibiose: aOR 1,53, 95 %-CI: 1,23–1,90
Pak T [26]	Vermutete ambulant erworbene Sepsis mit Schock (*n* = 25.990)	Schnellstmöglich	Pro h Verzögerung: aOR 1,07, 95 %-CI 1,04–1,11
Pak T [26]	Vermutete ambulant erworbene Sepsis ohne Schock (*n* = 23.619)	< 6 h	Verzögerter vs. früherer Beginn der Antibiose: aOR: 1,62, 95 %-CI: 1,26–2,08

*aOR* adjustiertes Odds Ratio, *CI* „confidence interval“

^a^Nach Diagnosestellung

Nach aktuellem Kenntnisstand sind somit anatomische Intervention wie auch Antibiose – bezogen auf den zeitlichen Abstand zur Diagnosestellung – eindeutig zeitkritisch. Aus dieser Erkenntnis heraus sollte auch das Zeitfenster zwischen dem Auftreten der ersten Symptome, deren Erkennung/Einordnung und dem Abschluss der Diagnostik prognostisch relevant sein. Um alle Zeitfenster so kurz wie möglich zu halten, ist die Kenntnis typischer, fachgebietsspezifischer Foci (Primärerkrankungen bzw. sekundäre Komplikationen), deren Symptome sowie fachgebietstypischer diagnostischer und therapeutischer Prozesse (einschließlich deren rasche Ingangsetzung) unerlässlich.

### Kernaussage 4.

Sowohl die anatomische Intervention wie auch die Antibiose sind – bezogen auf den zeitlichen Abstand zum Zeitpunkt der Diagnosestellung – eindeutig zeitkritisch. Um das Zeitfenster zwischen dem Auftreten der ersten Symptome, deren Einordnung und Diagnostik und dem Therapiebeginn so kurz wie möglich zu halten, ist die Kenntnis chirurgischer, fachgebietsspezifischer Inhalte unerlässlich.

## Die Bedeutung fachgebietsspezifischer Kenntnisse in der Intensivmedizin

Die Komplexität der kritischen Krankheit, die fachgebietsspezifischen Fokuscharakteristika (bezogen auf Ursache, Symptom und Diagnostik) sowie die notwendige zeitnahe Implementierung einer ursachengerechten Fokusdiagnostik und -therapie erfordern (zusätzlich zur Qualifikation für eine intensivmedizinische, organsupportive Therapie) ein Grundverständnis fachgebietsspezifischer Zusammenhänge und somit eine fundierte Aus- und Weiterbildung im jeweiligen Fachgebiet. Diese ist jedoch beim reinen Facharzt für Intensivmedizin nicht gegeben, beim Facharzt für fachspezifische Intensivmedizin (z. B. Facharzt für chirurgische Intensivmedizin) fraglich und nur erreichbar über den fachspezifischen Facharzt (z. B. für Chirurgie) mit konsekutiver intensivmedizinischer Zusatzweiterbildung.

Die Umwandlung der aktuell zur Verfügung stehenden intensivmedizinischen Zusatzweiterbildung (zu erwerben nach Abschluss der primären Facharztweiterbildung) in eine integrierte intensivmedizinische Weiterbildung (zu erwerben während der primären Facharztweiterbildung) und die gleichzeitige Beibehaltung (oder sogar Verkürzung) der Dauer der Facharztweiterbildung würden zu einer Reduktion der fachgebietsspezifischen Weiterbildungsinhalte während der Facharztweiterbildung führen. Damit würde auch eine neue Facharztweiterbildung für fachspezifische Intensivmedizin (z. B. Facharzt für chirurgische Intensivmedizin) geschaffen. Bei ggf. gleichzeitiger Reduktion der Gesamtweiterbildungsdauer halten wir im Interesse einer optimalen Patient:innenversorgung eine solche Umgestaltung für nicht sachdienlich. Eine überwiegende Konzentration auf intensivmedizinische Inhalte würde auch die gegenseitige Kommunikation und Zusammenarbeit zwischen verschiedenen Fachgebieten mit spezifischen Kenntnissen, Fachwissen und Fähigkeiten behindern. Die Einführung eines Facharztes für fachgebietsspezifische Intensivmedizin würde diese Kollegen auch von der übergreifenden Arbeit in dem jeweiligen Fachgebiet ausschließen und somit wertvolle Personalressourcen stilllegen.

Andererseits würde es die Beibehaltung des fachgebietsspezifischen Facharztes mit intensivmedizinischer Zusatzweiterbildung erlauben, dass Kolleg:innen weiterhin in ihr eigentliches Fachgebiet (temporär) zurückkehren können [28]. Ferner stellen bestimmte diagnostische Verfahren (Computertomographie, Endoskopie) und Therapien (Antibiose, radiologische Intervention) zur Fokuselimination kostenintensive Variablen dar, die einen signifikanten Anteil am intensivmedizinischen Budget besitzen (in Deutschland entfallen etwa 20 % der Krankenhauskosten auf die intensivmedizinische Versorgung, etwa 27 Mrd. € für 2022 [29–32]). Der Einsatz dieser Ressourcen kann nur dann mit einem adäquaten Aufwands-Ertrags-Verhältnis verbunden sein, wenn fachgebietsspezifische Kenntnisse zu den jeweiligen Indikationen und Kontraindikationen vorliegen.

Mit der Ablehnung eines Facharztes für fachgebietsspezifische Intensivmedizin stehen die Autoren dieser Arbeit nicht alleine. Bereits 2021 hat sich der multidisziplinäre gemeinsame Ausschuss für Intensivmedizin (Multidisciplinary Joint Committee of Intensive Care Medicine, MJCICM) der Europäischen Vereinigung der Fachärzte (European Union of Medical Specialists, UEMS) zu dieser Thematik geäußert – speziell auch als Reaktion auf die eingangs erwähnte Initiative der ESICM [28]. Die MJCICM hat diese Initiative als ungeeignet betrachtet, um die Intensivmedizin zu stärken, zumal sie ohne die Miteinbeziehung vieler wissenschaftlicher Gesellschaften ins Leben gerufen wurde, die sich ebenfalls mit Intensivmedizin befassen.

Ferner stimmten schon 2008 die in der Intensivmedizin tätigen Fachgebiete bei einem Treffen des MJCICM gegen die Idee, die Intensivmedizin zu einem unabhängigen Primärfach zu machen [33], und die Position des MJCICM hat sich seitdem nicht geändert. 2021 stellte das MJCICM fest, dass idealerweise die Weiterbildung in der Intensivmedizin mit der vorgeschalteten Qualifikation in einem primären Fachgebiet einhergehen sollte und dass diese fakultative Weiterbildung weiterhin nur zusätzlich zur Qualifikation in diesem Fachgebiet möglich sein sollte [28]. Die unter dem Dach der DGCH versammelten operativ tätigen Fachgebiete schließen sich dieser internationalen Einschätzung an und würden an folgenden Punkten festhalten:

## Fazit

1. Die intensivmedizinische Weiterbildung ist und bleibt integraler Bestandteil der chirurgischen Tätigkeiten, darf aber nicht zulasten fachgebietsspezifischer Weiterbildungsinhalte gehen.
2. Chirurgische, fachgebietsspezifische Kenntnisse sind Voraussetzung für eine effektive Therapie kritisch kranker Patient:innen. Eine frühe Einbindung und intensivmedizinische Erfahrung (im Rahmen der Facharztausbildung) der Weiterbildungsärzte muss allerdings gewährleistet sein, da die Altersstruktur und Komplexität der stationären Patient:innen immer weiter steigt und intensivmedizinische Kenntnisse und Fertigkeiten auch für die allgemeine perioperative Betreuung der Patient*innen dringend erforderlich sind. Diese Erfahrung im Rahmen der Facharztausbildung ist jedoch von der vertieften intensivmedizinischen Zusatzweiterbildung nach Abschluss der Facharztausbildung abzugrenzen und kann nur additiv zu dieser Zusatzweiterbildung erfolgen.
3. Die Zusatzweiterbildung Intensivmedizin muss auch in der nächsten Änderung der Weiterbildungsordnung weiter aus allen Facharztqualifikationen heraus gleichberechtigt erreichbar bleiben. Diese Forderung bezieht sich nicht nur auf alle Gebiete der Chirurgie, sondern auch auf Anästhesie, Neurologie und Innere Medizin. Dabei ist es aber gleichzeitig unabdinglich, dass diese Zusatzweiterbildung auf C1-Niveau verbleibt.

### Kernaussage 5.

Die intensivmedizinische Weiterbildung muss auch in der nächsten Änderung der Weiterbildungsordnung weiter aus allen Facharztqualifikationen heraus gleichberechtigt erreichbar sein und zwar in Form einer vollumfänglichen Zusatzweiterbildung, zu durchlaufen nach Abschluss der Facharztweiterbildung.
